# The mechanism of USP43 in the development of tumor: a literature review

**DOI:** 10.18632/aging.205731

**Published:** 2024-04-08

**Authors:** Ziqi Zhao, Meichen Liu, Zhikun Lin, Mengru Zhu, Linlin Lv, Xinqing Zhu, Rui Fan, Abdullah Al-Danakh, Hui He, Guang Tan

**Affiliations:** 1 Department of General Surgery, The First Affiliated Hospital of Dalian Medical University, Dalian Medical University, Dalian 116011, China; 2 Department of Neurology, The First Affiliated Hospital of Dalian Medical University, Dalian Medical University, Dalian 116011, China; 3 Department of Plastic Surgery, The First Affiliated Hospital of Dalian Medical University, Dalian Medical University, Dalian 116011, China; 4 Department of Pharmacy, The First Affiliated Hospital of Dalian Medical University, Dalian Medical University, Dalian 116011, China; 5 Department of Urology, The First Affiliated Hospital of Dalian Medical University, Dalian Medical University, Dalian 116011, China; 6 Institute of Cardiovascular Sciences and Key Laboratory of Molecular Cardiovascular Sciences, National, School of Basic Medical Sciences, Peking University, Beijing 100191, China; 7 Liaoning Key Laboratory of Molecular Targeted Drugs in Hepatobiliary and Pancreatic Cancer, Dalian 116000, China

**Keywords:** USP43, cancer, deubiquitinase, tumor immune microenvironment

## Abstract

Ubiquitination of the proteins is crucial for governing protein degradation and regulating fundamental cellular processes. Deubiquitinases (DUBs) have emerged as significant regulators of multiple pathways associated with cancer and other diseases, owing to their capacity to remove ubiquitin from target substrates and modulate signaling. Consequently, they represent potential therapeutic targets for cancer and other life-threatening conditions. USP43 belongs to the DUBs family involved in cancer development and progression. This review aims to provide a comprehensive overview of the existing scientific evidence implicating USP43 in cancer development. Additionally, it will investigate potential small-molecule inhibitors that target DUBs that may have the capability to function as anti-cancer medicines.

## INTRODUCTION

Most proteins undergo several modifications after synthesis, encompassing acetylation, methylation, phosphorylation, glycosylation, and ubiquitination [1–4]. Numerous signaling pathways are influenced by post-translational modification via the ubiquitination mechanism, which regulates target proteins’ location and/or activity. The ubiquitination process relies on three specific enzymes known as Ubiquitin enzymes (UBEs) that facilitate the covalent attachment of ubiquitin molecules to target proteins. Three key enzymes are ubiquitin-activating enzyme (E1), ubiquitin-conjugating enzyme (E2), and ubiquitin ligase or E3 ubiquitin ligase (E3). The target proteins first interact with E1 and E2, then selectively interact with E3 to regulate the specificity of substrates. Different forms of ubiquitination [5, 6], including monoubiquitination, polyubiquitination, and branching ubiquitination, are essential for intracellular activities and are engaged in various cellular functions [7], such as controlling the cell cycle and responding to DNA damage. Additionally, ubiquitination significantly affects many human diseases by regulating cell division, proliferation, and apoptosis [8, 9].

Ubiquitination is an essential process for both genesis and apoptosis; therefore, any disturbance in this process could potentially lead to cancer development [10–12]. Deubiquitinating enzymes (DUBs) may reverse the ubiquitination process of targeted proteins [13–18]. DUBs work by removing ubiquitin chains from their target proteins, which are vital for the stability or activity of the proteins [19, 20]. So far, there are about 100 kinds of DUBs in human genome [21], which can be divided into five families based on the organization of their catalytic domain [22]: (1) Ubiquitin-specific proteases (USPs), (2) ovarian tumor proteases (OTUs), (3) Ubiquitin C-terminal hydrolases (UCHs), (4) Machado-Joseph domain proteases (MJDs), and (5) JAB1/MNP/MOV34 metalloproteases (JAMMs).

**Figure 1:**
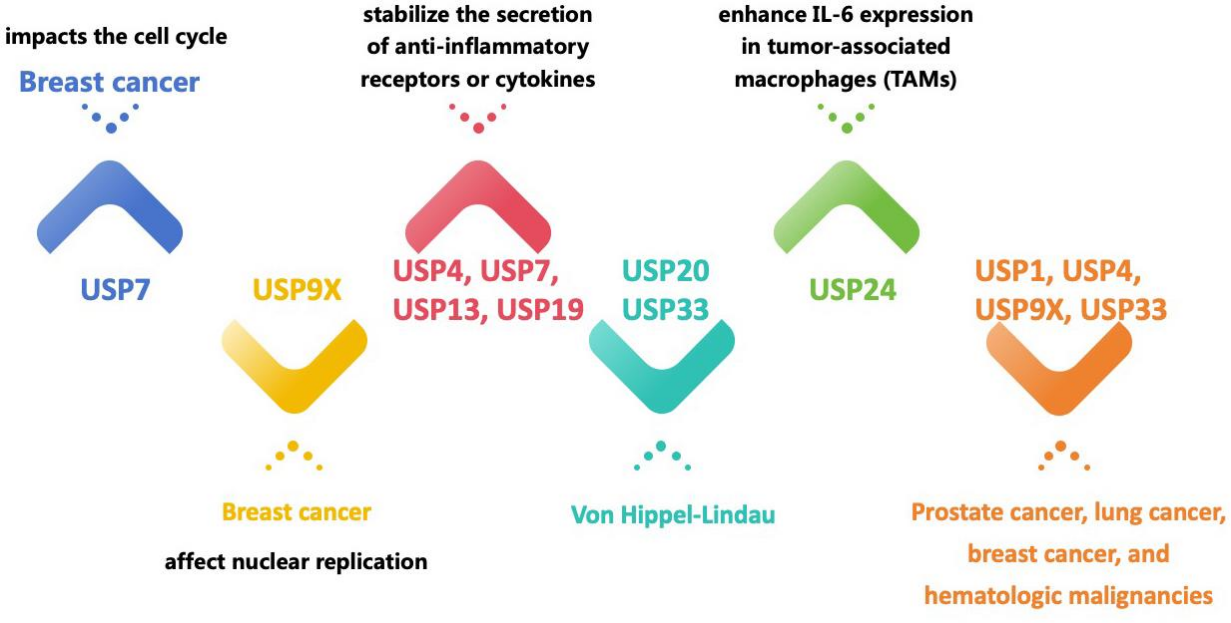
Different USPs regulate and promote the growth of cancerous cells and other diseases.

The USPs family is the most recognized group of DUBs, characterized by a wide range of structural and functional variability [23]. The distinguishing features of USPs include the unique catalytic core, known as the histidine and cysteine boxes, as well as the presence of zinc finger domains, ubiquitin-binding domains, or ubiquitin-like domains at the N and/or C-termini of the catalytic domain [21]. Protein degradation or oversynthesis is associated with malignancy’s development, spread, or growth. In this regard, a growing body of research has shown that USPs exert an influence on cancer progression through their ability to regulate and promote the growth of cancerous cells [12, 23, 24] (Figure 1). Previous studies have connected USPs, such as USP7 [25], USP21, USP22, USP33 [26], USP39, USP54 [27], and others, to carcinogenesis in several cancer types. While USP7 affects the cell cycle of breast cancer, which regulates its growth [28], USP9X influences breast cancer growth by influencing nuclear replication [29]. Additionally, USP9X controls mitosis, cell death, and treatment resistance in aggressive B-cell lymphomas [30]. Studies have indicated that the pathophysiology of Von Hippel-Lindau Syndrome (VHL) is linked to USP20 and USP33 [31–33]. The association between USPs and cell signaling pathways in malignancies have been demonstrated in previous studies.

Prominent signaling pathways implicated in the initiation and advancement of cancer, namely p53, NF-B, Wnt, and TGF-β, are impacted by the activities of USPs, including USP2, USP4, USP5, USP10, USP11, USP15, USP29, and USP34 [34]. Furthermore, USPs can control cytokine release patterns, with USP24 increasing IL-6 expression in tumor-associated macrophages (TAMs), whereas USP4, USP7, USP13, and USP19 can stabilize the release of anti-inflammatory receptors or cytokines [32–35]. It is worth mentioning that different types of cancer display divergent reactions to the identical USP protein. For instance, although USP24 inhibits the proliferation of tumor cells, it promotes the spread of metastases in other types of malignancies [4, 35]. In terms of treatment, USPs can be used as cancer therapeutic targets, such as inhibitors of USP1, USP4, USP7, USP9X, and USP33 for prostate cancer, lung cancer, breast cancer, and hematological malignancies, among others [26–30].

Nevertheless, the biological mechanisms of various proteins in the USPs family, such as USP43, remain unclear as limited research has been conducted on its role in malignancies. Published studies have explored the connection between USP43 and various types of cancer, including osteosarcoma, non-small cell lung cancer, lung squamous cell carcinoma, breast cancer, and colorectal cancer. These studies have shed light on the specific molecular processes and signaling pathways that are characteristic of each type of cancer.

## USP43 affects the proliferation and invasion of breast cancer

The involvement of USP43 in breast cancer has been documented in two previous studies suggesting that Ca_v_2.2 upregulates USP43 to promote tumorigenesis in breast cancer [36]. Ca_v_2.2 is an essential component of the voltage-dependent calcium channel family, which is responsible for regulating Ca^2+^ levels within physiological thresholds [37]. In support of this, the FDA has approved Ziconotide as a specific inhibitor of Cav2.2 for the treatment of chronic pain [38–41]. While Ca_v_2.2 expression is primarily restricted to brain tissue and absent in epithelial tissue, it has also been shown to be downregulated in normal breast epithelial cells and tissues, as opposed to an upregulation observed in breast cancer tissues and cell lines, particularly in triple-negative breast cancer (TNBC).

The extracellular matrix (ECM) plays a pivotal role in impeding the dissemination and metastasis of primary cancers, while certain cancer cells possess the capability to invade adjacent tissues through ECM degradation [42]. A specialized membrane structure with proteolytic activity is referred to as an invadopodium by which the invasive cells accomplish the invasion and metastasis. For instance, breast cancer cells utilize invadopodia to invade neighboring tissues and degrade the ECM [43]. The actin-binding protein cortactin plays a crucial role in the development of invadopodia [44]. Cortical actin also facilitates the synthesis and secretion of matrix metalloproteinases, which regulate ECM degradation [45, 46]. Similarly, Ca_v_2.2 enhances the production of invadopodia and the degradation of ECM, playing a crucial role in the dissemination and invasion of breast cancer. Further investigation into the underlying mechanisms of this procedure revealed that Ca_v_2.2 regulates USP43 synthesis through NFAT2 dephosphorylation, thereby facilitating the promotion of cortactin and subsequent growth of invadopodia, ultimately leading to metastasis in breast cancer. Addressing F-actin and actin regulatory proteins, which constitute the majority of invadopodia, poses a formidable challenge due to their intricate structural characteristics, rendering them an arduous therapeutic target. According to the aforementioned findings, USP43 exerts regulatory control over invadopodia development, thereby emerging as a promising therapeutic target for impeding invadopodia formation and attenuating the dissemination of breast cancer.

A subsequent investigation revealed a contrasting finding, suggesting that USP43 may function as a suppressive gene for breast cancer. The study proposed an imbalance in the inhibitory relationship between USP43 and EGFR/PI3K/AKT as a potential cause of cancer growth [47]. Numerous investigations have unequivocally established the pivotal role of the nucleosome remodeling and deacetylation (NuRD) complex in breast cancer development and metastasis [48–54]. Multiple subunits of the NuRD complex have been identified, and their expression varies depending on the type of cell and tissue. Histone H2BK120 can be deubiquitinated by the complex of USP43 and NuRD complex and be reactivated. It has been discovered that the USP43/NuRD complex inhibits the expression of numerous cancer-related genes, including the epidermal growth factor receptor (EGFR). EGFR mutations or amplifications are frequently associated with the progression of cancer and an unfavorable prognosis [55–59]. The PI3K-AKT pathway, strongly associated with cancer growth and prognosis, represents one of the signaling cascades that undergo hyperactivation upon EGFR activation [60–62]. The USP43/NuRD complex inhibits the activity of EGFR and AKT, while the EGFR/PI3K/AKT signaling pathway regulates the behavior of USP43. Phosphorylation by AKT enables USP43 to interact with the 14-3-3β/ε heterodimer and remain localized in the cytoplasm. The response inhibits the transcriptional function of USP43, while concurrently attenuating its negative regulation on the EGFR/PI3K/AKT signaling pathway. In line with this argument, a mutually inhibitory loop exists, the imbalance of which promotes breast cancer. These findings suggest that USP43 may serve as an essential component in the regulatory network of the EGFR/PI3K/AKT signaling pathway [47]. Therefore, it has been established that the USP43/NuRD complex inhibits the growth, invasion, and metastasis of breast cancer cells; thus, USP43 may serve as a suppressor of breast cancer.

## USP43 affects the proliferation and invasion of colorectal cancer

As a member of the zinc finger E-box (ZEB) family of transcription factors, ZEB1 plays a crucial role in regulating cell differentiation [63], especially in controlling epithelial-mesenchymal transformation (EMT), which is vital for cancer progression. Aberrant expression of ZEB1 has been detected in a wide range of cancer types, such as cervical cancer, pancreatic cancer, osteosarcoma, lung cancer, liver cancer, stomach cancer, colorectal cancer, and breast cancer [64]. According to this study [65], The expression of USP43 is significantly upregulated in colorectal cancer and correlates with an unfavorable prognosis. During the investigation into the role of USP43 in the pathogenesis of colorectal cancer, it has been revealed that USP43 exhibits a capacity to enhance *in vitro* proliferation, migration, and invasion of colorectal cancer cells. Further investigation revealed a direct interaction between USP43 and ZEB1 in colorectal cell lines, wherein USP43 downregulates ZEB1 protein expression independent of its transcriptional regulation, thereby modulating the abundance of ZEB1 protein. Consequently, USP43 exerts an influence on the process of ubiquitination, thereby affecting the degradation of ZEB1 protein. Moreover, modulation of USP43 expression or knockdown in combination with ZEB1 knockdown or overexpression can exert an impact on various EMT-related biomarkers including E-cadherin, N-cadherin, and vimentin, as well as cell invasion and migration. Therefore, it demonstrates that USP43 and ZEB1 govern the regulation of EMT, which plays a pivotal role in initiating and advancing colorectal cancer.

ZEB1 exerts a significant impact on not only the formation and progression of malignancies but also the development of resistance to chemotherapy. For instance, the knockdown of ZEB1 reduces both the invasiveness of glioblastoma cells and their susceptibility to the chemotherapeutic agent temozolomide [66]. The expression of ZEB1 is strongly associated with the resistance of cancer cells to chemotherapeutic agents including gemcitabine, 5-fluorouracil, and cisplatin in pancreatic cancer cells. Furthermore, the impact of USP43 on the susceptibility of colorectal cancer cells to chemotherapy, reveals a potential role for USP43 in upregulating ZEB1 expression and downregulating chemotherapy resistance in colorectal cancer.

Therefore, USP43 emerges as a promising therapeutic target for the management of colorectal cancer, implying that the aberrant functioning of USP43 may contribute to the progression of this malignancy.

## USP43 affects the proliferation and prognosis of pancreatic ductal adenocarcinoma (PDAC)

The majority of pancreatic cancer cases, approximately 90%, are attributed to PDAC, which unfortunately has a low 5-year survival rate of only 8% to 10% [67, 68]. Moreover, a significant proportion of patients with PDAC who are diagnosed with Stage IV generally pass away within a year. As a result of the considerable biological diversity and aggressiveness demonstrated by PDAC, in addition to the broad spectrum of patient prognoses and therapeutic responses, the current treatment options are quite limited in scope [69]. Immunotherapy has recently made significant advancements in the treatment of various malignancies, such as melanoma and breast cancer [70–73], which have been demonstrated to significantly extend cancer survival. Nevertheless, the potential of PDAC for immunotherapy is constrained by three key factors. Firstly, the unique tumor immune microenvironment (TIME) associated with PDAC poses a physical barrier that hampers immune system functionality, impeding the identification and elimination of PDAC by immune cells [74, 75]. Secondly, the majority of immune cells that have infiltrated the peritumoral microenvironment in PDAC are immunosuppressive cells [76, 77]. Thirdly, the TIME of PDAC may be impacted by the intricate network between cytokines and cytokine receptors [78], thereby promoting cancer progression. Consequently, PDAC is considered an “immune desert” or an immunologically “cold tumor”.

A recent study reports that USP43 is significantly upregulated in PDAC, suggesting its potential role in influencing tumor formation by promoting the proliferation of the expression of USP43 in PDAC [79]. Additionally, a positive correlation has been observed between elevated levels of USP43 expression and an unfavorable prognosis in patients with PDAC. These findings suggest that USP43 may serve as a pivotal regulator of PDAC proliferation and hold potential as an independent prognostic indicator. Further analysis of USP43 expression in PDAC revealed a negative association with the chemokine signaling pathway and cytokine-cytokine receptor interactions. The complex interaction of cytokines and receptors can have a substantial influence on PDAC TIME, potentially resulting in immune evasion and faster malignant development. As a result, identifying targets that modify TIME in PDAC, moving it from a “cold” to a “hot” state, has the potential to improve PDAC’s responsiveness to immune checkpoint inhibitors. An additional study of the TIME surrounding PDAC has shown an inverse relationship between USP43 expression and CD8^+^ T cell activation, indicating that USP43-mediated suppression of CD8^+^ T cell infiltration leads to a poor prognosis [80–83].

## USP43 affects the proliferation and invasion of lung cancer

Lung cancer, which is the most common cancer type among other organs, has a poor prognosis, and scientists are interested in finding new modes of treatment, one of which is ubiquitination. A recent study established a correlation between USP43 and lung squamous cell carcinoma (LUSC), indicating that USP43 facilitated LUSC proliferation and invasion, indicating an unfavorable prognosis [84]. In patients with LUSC, increased USP43 expression is substantially correlated with tumor invasiveness, including larger tumors and advanced TNM stages, and with a reduced overall survival rate. Notably, variability in the expression of USP43 within LUSC tissues is observed among patients. The results of their study provide support for the claim that USP43 has the potential to function as an innovative and autonomous prognostic indicator that can forecast survival outcomes. The *in vitro* investigations have shown that USP43 stimulates the growth and spread of LUSC, which is consistent with its reported influence on breast and colorectal cancers [65, 85]. The expressions of cyclin-dependent kinase 1 (CDK1), vimentin, and snail are shown to be downregulated after USP43 knockdown, giving more evidence for USP43’s carcinogenic function in LUSC. As a result, the downstream ramifications of USP43 were studied; nevertheless, the specific underlying mechanism remains unknown to the researchers, emphasizing the need for more study. The data presented above suggest that USP43 is a unique prognostic biomarker for patients with LUSC. Furthermore, its deletion has a significant anti-cancer impact by efficiently reducing the proliferation and invasion of LUSC [84].

A further investigation [86] was undertaken to construct a radiation prediction model for early-stage or locally progressed non-small cell lung cancer (NSCLC) using six genes, including APOBEC3B, GOLM1, FAM117A, KCNQ1OT1, PCDHB2, and USP43. The high-risk group for those genes had considerably lower overall survival (OS) and progression-free survival (PFS) than the low-risk group; however, this study did not include a comprehensive examination of individual genes. In conclusion, USP43 is a predictive gene in NSCLC patients, particularly LUSC patients, and further research with large cohorts is needed to explain its specific involvement.

## USP43 affects the proliferation and invasion of osteosarcoma

In investigating the role of USP43 in osteosarcoma [87], it was observed that patients with osteosarcoma displayed high levels of USP43, which contributes to the regulation of EMT, a critical cellular mechanism implicated in the progression of osteosarcoma. Furthermore, the upregulation of EMT transcription factors such as Snail, ZEB, or Twist promotes the production of EMT. Notably, elevated expression levels of ZEB1 were observed in osteosarcoma tissue compared to healthy bone tissue. In patients with osteosarcoma and lung metastasis, USP43 deubiquitinates ZEB1 and maintains its transcription [65], resulting in increased invasion of osteosarcoma cells via inducing EMT [88, 89].

## USP43 promotes glycolysis and metastasis in bladder cancer (BLCA)

The primary factors contributing to the unfavorable prognosis of BLCA are invasion and recurrence [90]. Under aerobic conditions, cancer cells obtain energy via glycolytic metabolism rather than oxidative phosphorylation; this process is known as the Warburg effect [91]. Warburg effect is closely related to the pathogenesis and aggressiveness of BLCA [92]. c-Myc plays a crucial role in regulating aerobic glycolysis [93]. It has been observed that BLCA induces amplification of the MYC oncogene, and its products may contribute to the tumorigenesis of BLCA [94–96]. Nevertheless, c-Myc is prone to ubiquitase degradation, thereby impeding tumor progression [97–100].

A recent study [101] demonstrates that USP43 is substantially upregulated in BLCA and that its expression increases with tumor grade. Moreover, USP43 may stimulate the metastasis of BLCA. The examination of differentially expressed genes revealed a positive correlation between USP43 and both the glycolysis pathway and the MYC target pathway. As a result, the researchers conducted additional investigations into the correlation between USP43 and c-Myc and discovered that USP43 deubiquitinated c-Myc at K148 and K289, thereby stabilizing its expression. c-Myc oncoprotein promotes the transcription of USP43 and regulates the transcription of at least 15% of the entire genome as a transcription factor. Consequently, USP43 and c-Myc form a feedback loop in which their activity is reciprocal; an asymmetry in this loop results in atypical glycolysis and c-Myc accumulation, both of which initiate the malignant behavior of BLCA. Furthermore, therapeutic targeting of c-Myc is challenging due to its localization and reaction affinity within the nucleus. In summary, USP43 emerges as a prospective therapeutic target for BLCA.

## USP43 promotes the proliferation of epithelial ovarian cancer (EOC) and impairs its cisplatin sensitivity

Epithelial ovarian cancer (EOC) is the predominant form, representing over 90% of all ovarian cancer cases, with the majority being diagnosed in advanced stages [102–104]. Primary treatments for EOC include debulking surgery and a combination of cisplatin and taxane chemotherapy [105]. Cisplatin administration frequently leads to drug resistance, impacting the effectiveness of chemotherapy, so it is crucial to identify new effective treatment targets [106, 107].

Previous research discovered that USP43 acts as a promoter in various types of cancer, including EOC. According to a study [108], the cancer’s malignant characteristics are more pronounced in patients with high levels of USP43 in EOC. High levels of USP43 in EOC are linked to a poor prognosis. The study delves into the tumor-promoting impact in EOC, showing that USP43 enhances the proliferation, invasion, and migration of EOC, and facilitates EOC cells to enter the cell cycle’s proliferation phase.

Cisplatin is a primary chemotherapeutic treatment for EOC. Researchers investigate the impact of USP43 on the efficacy of cisplatin in EOC cells that are resistant to the drug. USP43 suppressed DNA damage and apoptosis, thereby reducing the sensitivity of EOC cells to cisplatin. Genes associated with USP43 in EOC primarily focus on controlling cancer advancement and histone deacetylation (HDAC). HDAC2, a member of the HDAC family, is shown to be significantly upregulated in EOC, and its high expression is linked to a negative prognosis. The same study also explores the correlation between USP43 and HDAC2. USP43 is discovered to remove ubiquitin from HDAC2, leading to the stabilization of the HDAC2 protein. USP43 hindered the responsiveness of EOC cells to cisplatin by targeting HDAC2. Upon further investigation, it was discovered that HDAC2 triggers the Wnt/β-catenin pathway, leading to decreased sensitivity of EOC cells to cisplatin.

Overall, USP43 affects the susceptibility of EOC cells to cisplatin and is identified as a therapeutic target to overcome cisplatin resistance.

## Conclusion and future perspectives

TP53 is the most important cancer-related gene. This tumor suppressor gene, found on chromosome 17p13.1, is linked to both hereditary and sporadic malignancies. Mutations in the TP53 gene, which affects 17p13.1 of the human genome, are among humans’ most common genetic alterations and are thought to represent hereditary malignant transformation [109–111]. Interestingly, the presence of the USP43 gene in this chromosomal region implies that mutations in 17p13.1, particularly deletions, may have a negative impact on USP43 functioning. Notably, online database studies revealed frequent alterations and/or lack of USP43 in numerous types of cancer, thereby compelling further investigation into its function in the progression of cancer and potential targets as a novel therapeutic approach.

**Figure 2:**
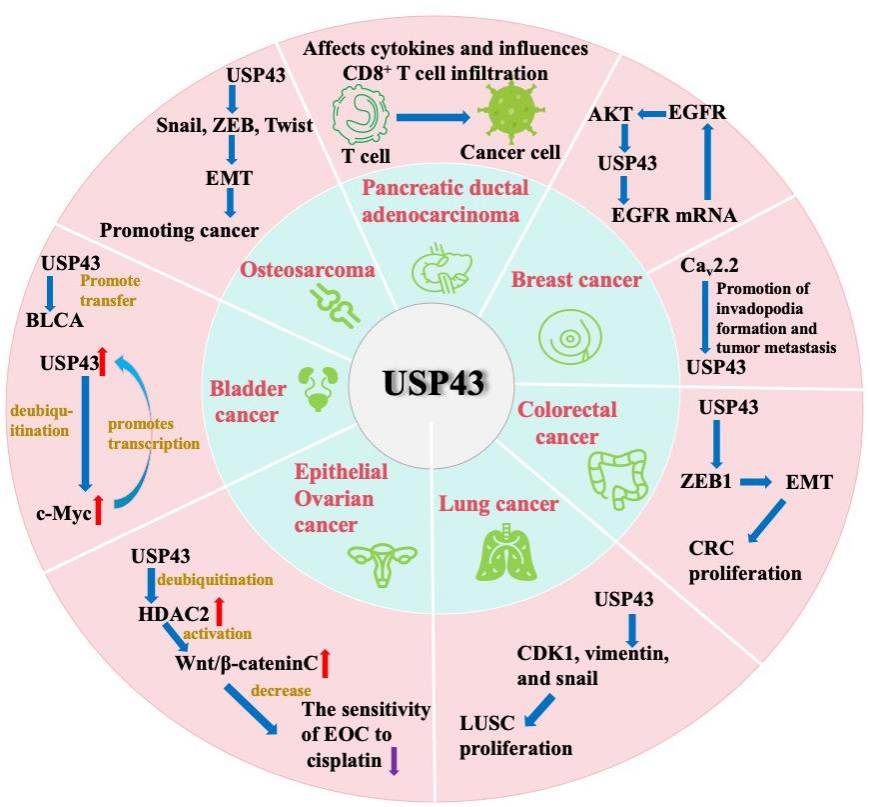
The potential significance of USP43 across diverse cancer entities.

Studies in databases and clinicopathological specimens have confirmed that USP43 expression is significantly elevated in breast, pancreatic, lung, bladder, and epithelial ovarian cancers. Furthermore, the USP43 level is associated with poor prognosis and its involvement in different cancer pathways. USP43 potentially regulates cancer invasion by modulating EMT-associated transcription factors, such as ZEB1 or SNAIL, in breast cancer, colorectal cancer, and osteosarcoma. In breast cancer, USP43 promotes invasion and metastasis through invadopodia formation. In BLCA, USP43 promotes glycolysis and metastasis. In EOC, USP43 promotes the proliferation and impairs cisplatin sensitivity. Moreover, it may govern the duration of PDAC progression while facilitating CD8^+^ T cell infiltration. Additionally, USP43 can be employed in conjunction with other genes to predict lung cancer outcomes. Therefore, USP43 not only affects the proliferation and metastasis of tumors directly but also influences the immune microenvironment of some kinds of cancers, which affects their biological behavior (Figure 2 and Table 1).

**Table 1:** The prognostic significance of ubiquitin-specific protease 43 (USP43) across diverse cancer entities.

**Cancer**	**Pathway**	**Year**
Breast cancer	Cav2.2-NFAT2-USP43 axis facilitates invadopodia formation and metastasis [36].	2022
	Imbalance of the reciprocally inhibitory loop between the USP43 and EGFR/PI3K/AKT drives breast carcinogenesis [47].	2018
Pancreas	Modulating the proliferation and infiltration of surrounding immune cells [79].	2023
Lung cancer	Promoting growth and invasion of LUSC may relate to CDK1, vimentin, and snail [84].	2022
	Predicting the risk of NSCLC with the other five genes [86].	2022
Colorectal cancer	Regulating ZEB1 protein and mediating proliferation and metastasis [65].	2021
Osteosarcoma	Regulating EMT and progression [87].	2021
Bladder cancer	USP43 stabilizes c-Myc, and c-Myc promotes the translation of USP43. This loop imbalance between USP43 and c-Myc drives glycolysis and bladder carcinogenesis [101].	2024
Epithelial ovarian cancer	The stabilization of HDAC2 by USP43 and the modulation of the Wnt/β catenin signaling pathway result in a reduction in cisplatin sensitivity [108].	2024

In conclusion, our findings demonstrate a high frequency of USP43 mutations across various human cancers, implicating its pivotal role in tumorigenesis and prognosis prediction for chemo- and radiotherapy efficacy. USP43 may be a potential indicator for predicting the prognosis of cancer and may also be a conducive target for monitoring cancer therapy in the future. These results highlight the potential of targeting USP43 as a promising therapeutic strategy for future cancer treatment.
